# An Intelligent Maneuver Decision-Making Approach for Air Combat Based on Deep Reinforcement Learning and Transformer Networks

**DOI:** 10.3390/e26121036

**Published:** 2024-11-29

**Authors:** Wentao Li, Feng Fang, Dongliang Peng, Shuning Han

**Affiliations:** 1 School of Automation, Hangzhou Dianzi University, Hangzhou 310018, China; lwt_super@163.com (W.L.); dlpeng@hdu.edu.cn (D.P.); 2 China Academy of Launch Vehicle Technology, Beijing 100076, China; jay34220513@163.com

**Keywords:** air combat maneuver decision-making, deep reinforcement learning, transformer network, information loss environment

## Abstract

The traditional maneuver decision-making approaches are highly dependent on accurate and complete situation information, and their decision-making quality becomes poor when opponent information is occasionally missing in complex electromagnetic environments. In order to solve this problem, an autonomous maneuver decision-making approach is developed based on deep reinforcement learning (DRL) architecture. Meanwhile, a Transformer network is integrated into the actor and critic networks, which can find the potential dependency relationships among the time series trajectory data. By using these relationships, the information loss is partially compensated, which leads to maneuvering decisions being more accurate. The issues of limited experience samples, low sampling efficiency, and poor stability in the agent training state appear when the Transformer network is introduced into DRL. To address these issues, the measures of designing an effective decision-making reward, a prioritized sampling method, and a dynamic learning rate adjustment mechanism are proposed. Numerous simulation results show that the proposed approach outperforms the traditional DRL algorithms, with a higher win rate in the case of opponent information loss.

## 1. Introduction

With the rapid development of uncrewed aerial vehicle (UAV) technology and autonomous decision-making approaches, the characteristics of air combat including fast pace, strong adversarial games, and incomplete information have been presented [1]. For autonomous maneuver decision-making in air combat, UAVs use specific methods such as optimal control, differential game, and machine learning to generate maneuvering commands based on the environment situation (including opponent information) acquired by its onboard detection equipment [2]. Autonomous maneuver decision-making is significant to dogfighting. The autonomous maneuver decision-making method is key to winning dogfights since it leads UAVs to occupy an advantageous position. How to use the obtained air combat situation to generate decision-making commands accurately is currently the difficulty of air combat maneuver decision-making [3]. Thus, it is important to investigate different intelligence decision-making methods to improve the decision-making speed and quality of UAVs.

The autonomous maneuver decision-making approaches in air combat include mathematical, search-based, and data-driven approaches. For the mathematical approach, the maneuver decision-making problem is formulated as an optimization problem and solved by using mathematical analytical methods, such as differential games [4,5], the bi-objective optimization method [6], and the situational function optimization method [7]. Although the analytical solutions obtained from these methods are clear, the calculations can be quite complex. For the search-based approach, the maneuver decision-making problem is modeled as a discrete variable optimization problem, and it is solved by matrix decision-making [8], heuristic algorithms [9], and dynamic programming [10]. However, for these search-based approaches, finding a satisfactory solution within a finite number of iterations becomes challenging as the problem size increases. For the data-driven approach, it includes neural networks [11], fuzzy algorithms [12,13], and reinforcement learning [14,15]. The data-driven approach modeled the maneuver decision-making problem as a mapping problem between different air combat situations and maneuver decision commands.

Reinforcement learning is a good option for solving sequential decision problems, which gives agents the ability to perform self-supervised learning. In order to obtain the maximum accumulated reward, the agent interacts with the environment and continuously adjusts its own strategy by obtaining reward in the environment. Deep reinforcement learning (DRL) combines deep neural networks and reinforcement learning approaches, utilizing the powerful representation and mapping capabilities of neural networks to approximate the reward function of state and action and map from state to action. DRL is an effective approach to solving high-dimensional state–action problems, and it is widely used in fields such as electronic games, recommendation systems, and intelligent control. A parameter-shared Q-network (PS-DQN) method is proposed in Reference [16]. Multi-UAV maneuver decision-making applies PS-DQN to converge the strategy to Nash equilibrium with virtual self-play. But it is assumed that each UAV is flying at the same altitude and can detect the accurate position of enemy UAVs within a certain range. In Reference [17], the Soft Actor Critic (SAC) approach is used to design the maneuver decision-making algorithm. Compared with the Twin Delayed Deep Deterministic Policy Gradient (TD3) algorithm, the simulation results show that the SAC algorithm has a shorter training time and a higher win rate. In Reference [18], a one-to-one air combat model and missile attack zone are built, and then a Parallel Self-Play training SAC algorithm (PSP-SAC) is proposed for sharing the sample and policy in multiple combat environments. In Reference [19], an Asynchronous Advantage Actor–Critic (A3C) algorithm is proposed, employing a multi-threaded asynchronous mechanism to reduce input correlation and accelerate training compared to methods based on experience replay. In Reference [20], a bidirectional recurrent neural network (BRNN) is used to achieve communication between UAV individuals, and the multi-UAV cooperative air combat maneuver decision model under the actor–critic architecture is established. The decision model can obtain the cooperative maneuver policy through reinforcement learning, and guide UAVs to obtain the overall situational advantage and defeat the opponents under tactical cooperation. In Reference [21], a maneuver decision-making approach is proposed by applying the LSTM network to the actor and critic networks in the PPO method, which has the ability to learn temporal air combat data. The simulation results show that this approach can improve the decision-making agent’s learning efficiency and decision quality. In Reference [22], the DRL-based maneuver decision-making approach is proposed by using the LSTM-Dueling DQN network, and the simulation results show that the agent training efficiency is improved. In Reference [23], an intelligent maneuver planning method for Beyond-visual-range (BVR) air combat using an improved deep Q network (DQN) based on the LSTM network is proposed; the results show that agents can effectively avoid enemy threats and gain tactical advantages. In Reference [24], a data-driven approach using the LSTM neural network is proposed to predict missile trajectories in a model-free manner, leveraging their capacity to learn long-term temporal dependencies and handle both measurement noise and motion uncertainties.

The Transformer network achieves great success in dealing with time series data [25], and introducing it into DRL has become a current research hotspot. In [26], the self-attention mechanism in the Transformer network is introduced in DRL for structured state representation inference. In [27], self-attention is applied to representation learning, which extracts relationships between multiple agents to learn and express strategies better. In [28], the Transformer architecture is introduced to offline reinforcement learning, which is directly applied to the sequence decision-making model.

According to the above references, the following limitations have been identified. Firstly, the completely observable air combat environment is assumed in a variety of approaches, and the situation between the enemy and our agent is assumed to be completely available. However, in real application cases, the environment and agent state information is hard to obtain, and usually only partial information can be obtained. Secondly, a fully connected neural network is applied to DRL algorithms in most approaches. The fully connected network has a simple and intuitive structure with strong data mapping ability, but it is difficult to capture the dependency relationships in temporal data. The LSTM network is applied in some approaches [19,29], which use the gating mechanism to process data with temporal dependencies and prevent gradient vanishing effectively. Compared to fully connected networks, the dependency relationships between sequence inputs can be captured by the LSTM network. Compared to traditional RNNs and GRUs, additional gating units and memory units are introduced to solve the problem of gradient vanishing during propagation. However, the data are input in a serial manner, and the gating mechanism requires a large amount of computation, resulting in high training time costs. Thus, the DRL with fully connected networks and recurrent neural networks cannot work well in the situation of enemy information loss. Thirdly, although the Transformer network has been introduced into DRL, its application to air combat decision-making has not been sufficiently studied. Thus, the above approaches [16,17,18,19,20] cannot provide reliable performance for cases with incomplete information in air combat decision-making, especially for cases with opponent information loss. Therefore, according to the above analysis, it is important to investigate the intelligent maneuver decision-making method in the information loss case to improve the decision-making quality. 

In this paper, the Transformer network is introduced to design the actor and critic networks in DRL architecture. Considering the maneuver decision-making problem in air combat is a continuous-variable optimization problem, the Deep Deterministic Policy Gradient (DDPG) method based on the framework of actor and critic performs well to deal with this problem. Thus, in this paper, an approach based on DDPG combined with a Transformer network is proposed. The main contributions of this paper are concluded as follows:

(1) Considering the information loss, the Transformer network is introduced to design the actor and critic networks in DDPG, which can extract hidden relationships in the temporal trajectory samples and use this relationship as the reference for the agent network when opponent information is unavailable. Then, the maneuver decision quality can be improved in the information loss situation.
(2) The issues of limited experience samples, low sampling efficiency, and poor stability appear when the Transformer network is introduced into the DDPG algorithm. To address the issue of limited experience samples, a more effective reward function is designed by combining the global and local rewards, and meanwhile, an exploration mechanism is designed, which encourages the agent to explore the decision-variable space thoroughly and accumulate a large number of various experience samples. To address the issue of low sampling efficiency, a prioritized sampling mechanism is designed in the episode experience replay, assigning higher sampling probabilities to focus on more informative episodes and improve training efficiency. To address the issue of poor stability, a dynamic learning rate adjustment mechanism is designed to make a quick rapid gradient descent in the initial training stage and an accurate parameter setting in the later training stage.
(3) Based on numerous experiments, the proposed approach has a better performance in the case of a 10% probability of information loss compared with the traditional DDPG-based decision-making approach. This result proves the effectiveness of the proposed maneuver decision-making approach.

## 2. Air Combat Environment Model for DRL

The air combat environment model is first developed to make sure the DRL algorithm can be trained. It contains the UAV kinematic model, the UAV sensors and weapon capabilities simulation, and the rules of victory judgment.

### 2.1. UAV Kinematic Model

**Figure 1:**
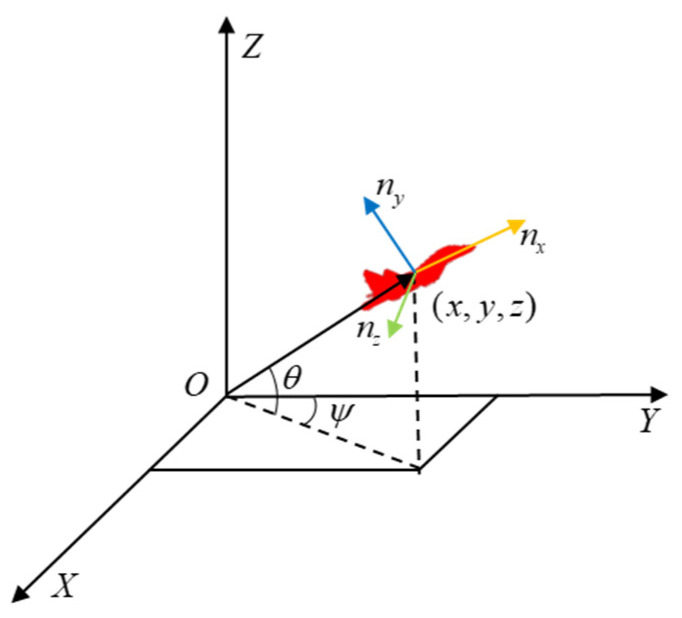
The UAV in the inertial coordinate system.

The UAV in the inertial coordinate system is treated as a mass point as shown in Figure 1, ignoring the effects of angle of attack and sideslip on the UAV. The kinematic model of the UAV is established as follows:

(1) $$\left\{\begin{array}{l} \dot{x}=V\cos\theta \cos\psi \\ \dot{y}=V\cos\theta \sin\psi \\ \dot{z}=V\sin\theta \\ \dot{V}=g(n_{x}-\sin\theta )\\ \dot{\theta }=g(n_{z}-\cos\theta )/V\\ \dot{\psi }=gn_{y}/V\cos(\theta ) \end{array}\right.,$$

where V represents the speed of the UAV, and $\dot{x},\;\dot{y},\;\dot{z}$ represents the velocity component along the coordinate axis. θ and ψ represent the pitch and yaw, respectively, and g represents the gravitational acceleration. $n_{x}$, $n_{y}$, and $n_{z}$ are the tangential, lateral, and normal overload, respectively. Based on Equation (1), the Runge–Kutta method is applied to compute the trajectory of the UAV with a simulation step of $t_{s}$, which is given by the initial state.

### 2.2. Rules of Victory Judgment for Dogfight Simulation

The one-to-one close-range air combat (i.e., dogfight) scenario is considered. It is assumed that the red UAV is its own side aircraft and the blue UVA is the opponent aircraft. Meanwhile, both UAVs are the same, with the same sensors and weapon capabilities and identical maneuverability. The red UAV employs the deep reinforcement learning algorithm proposed in this paper for maneuver decision-making, while the blue side’s UAV employs a fusion of matrix game and genetic algorithms to make the maneuver decision. The goal of maneuver decision-making is to make the UAV occupy the advantageous attack position, where the opponent UAV is located in the weapon’s attack range for a fixed period of time to guarantee successful shooting.

**Figure 2:**
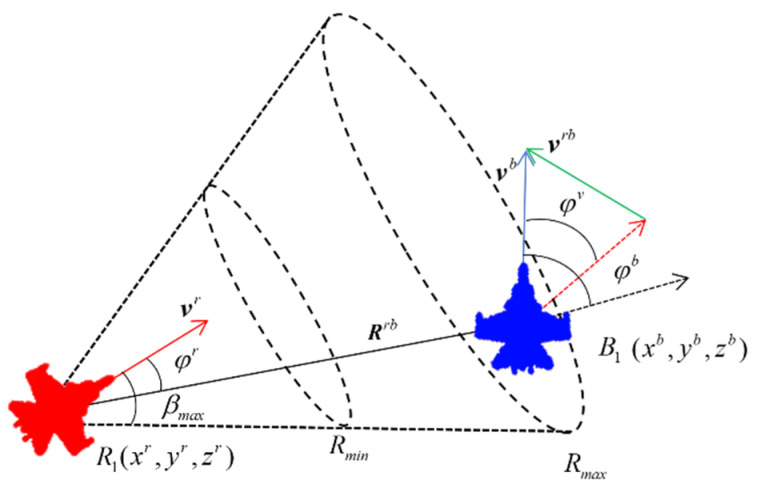
The relative positions of uncrewed aerial vehicles in air combat.

The relative positions of UAVs in dogfights are illustrated in Figure 2, where $\left(x^{r},y^{r},z^{r}\right)$ and $\left(x^{b},y^{b},z^{b}\right)$ represent the position coordinates of the red and blue UAVs, respectively. $v^{r}$ and $v^{b}$ are the velocity vectors of the red and blue UAVs, with minimum and maximum speeds denoted as $v_{\min}$ and $v_{\max}$, respectively. $v^{rb}$ is the relative velocity vector, and $\varphi^{v}$ is the angle of velocity vectors. $R^{rb}$ is the relative distance vector, and $\varphi^{r},\varphi^{b}$ represent the velocity leading angles. $R_{\max}$ and $R_{\min}$ are the maximum and minimum attack distances of UAV weapons, and $\beta_{\max}$ represents the maximum attack angle of the weapon.

In the air combat simulation, the rule of victory judgment is made as follows: when one side’s UAV locks on the other side’s UAV in its weapon attack zone for a period of time, then it is assumed to win the game. The victory condition is described as follows:

(2) $$\left\{\begin{array}{l} \varphi^{r}\leq \beta_{max}\\ R_{min}\leq |R^{rb}|\leq R_{max}\\ t_{lock}\geq t_{p} \end{array}\right.,$$

where $t_{p}$ is the set lock-on time. Moreover, if neither side’s UAV satisfies the victory condition in the maximum game simulation time, $t_{\max}$, the dogfight round is considered a draw.

### 2.3. State–Action Pair Design for DRL

The state vector of DRL at time instant *t* is defined as Equation (3), which includes information on relative distance, relative velocity, and associated angles as follows:

(3) $$s_{t}=[R^{rb},v^{rb},\varphi^{v},\varphi^{r},\varphi^{b}],$$

where each component of the state vector can be computed as follows:

(4) $$\left\{\begin{array}{c} R^{rb}=[x^{b}-x^{r},y^{b}-y^{r},z^{b}-z^{r}]\\ v^{rb}=v^{b}-v^{r}\\ \varphi^{v}=\arccos(\frac{v^{r}\;v^{b}}{\left|v^{r}|\;|v^{b}\right|})\\ \varphi^{r}=\arccos(\frac{v^{r}\;R^{rb}}{\left|v^{r}|\;|R^{rb}\right|})\\ \varphi^{b}=\arccos(\frac{v^{b}\;R^{rb}}{\left|v^{b}|\;|R^{rb}\right|}) \end{array}\right..$$

Considering the factors of electronic jamming or quick evasion maneuvering, the UAV may fail to obtain information about the opponent UAV such as position, velocity, and flight path angle. In order to simulate this scenario of information loss, we set the probability of losing target information during air combat as $p_{loss}=10\%$ and let the state vector be null.

Based on the kinematic model in Equation (1), the UAV is controlled by three accelerations in its body coordinate system. Therefore, the decision-making action for DRL at time instant *t* is defined as follows:

(5) $$a_{t}=[n_{x},n_{y},n_{z}],$$

where each acceleration component should satisfy the overload saturation constraint denoted as follows:

(6) $$n_{i,\min}\leq n_{i}\leq n_{i,\max},\;\left(i=x,y,z\right).$$

## 3. Opponent UAV Decision-Making Model Based on the Genetic Algorithm Optimizing Matrix Game

It is important to build a competitive UAV opponent for training the DRL-based UAV decision-making model. Here, a maneuver decision-making approach is adopted, which combines a genetic algorithm with a matrix game to generate opponent UAV actions. A matrix game is often used to solve zero-sum game problems, where the decision-maker can only choose an optimal one from the finite strategy set. In order to use the matrix game, the common basic maneuvering command set is built and both UAVs are assumed to select a command from the library. The basic command set consists of seven maneuvers: maintaining the current state, maximum overload acceleration/deceleration, left/right turns, pull-up, and dive. 

### 3.1. Maneuver Decision-Making Using the Matrix Game Method

The matrix game method is based on game theory knowledge and is used to solve two-person finite zero-sum game problems, where decision-makers can only choose from a limited set of strategies during the decision-making process. The blue side uncrewed aerial vehicle uses a genetic algorithm to optimize the maneuvering strategy generated by matrix countermeasures and uses it as an air combat maneuver decision-making method to enable the uncrewed aerial vehicle to quickly form an attack advantage on the target. Firstly, the instructions in the typical maneuver instruction set are used as candidate decisions. Then, matrix games are used to estimate the strategies of the agent and the opponent to select specific decision instructions as candidate optimal actions. Next, genetic algorithms are used to iteratively optimize and search for the optimal action instructions in the neighboring domains within the candidate optimal action space, further improving decision quality while ensuring decision speed.

Assuming both sides employ one of the seven typical maneuvering actions, the advantage value of the blue side over the red side can be calculated after one decision step. In this scenario, the red side selects the *n* th maneuvering method, while the blue side selects the *m* th maneuvering method. By traversing all the action sets of both the red and blue sides, the advantage matrix for the blue side can be calculated as follows:

(7) $$A_{m,n}=\left(\begin{array}{llll} adv_{11} & adv_{12} & \ldots & adv_{17}\\ adv_{21} & adv_{22} & \ldots & adv_{27}\\ \vdots & \vdots & adv_{mn} & \vdots \\ adv_{71} & adv_{72} & \ldots & adv_{77} \end{array}\right).$$

To ensure that the blue side selects a robust decision value, where the chosen action maximizes its overall advantage after maneuvering regardless of the maneuvering strategy adopted by the red side, the blue side should select the action A* corresponding to the row sum’s maximum value in the advantage matrix as the decision result.

### 3.2. Genetic Algorithm

The maneuver strategy optimized using the above matrix game method is a coarse-grained approach with low control accuracy, indicating that there is still room for improvement in the acceleration decision. Therefore, based on the matrix game framework, a genetic algorithm is integrated into the matrix game framework to select action values as the central reference for determining decision values. An optimization interval is established at 0.1*g*, extending 1*g* in both the overloaded left and right directions. Through continuous iteration, the genetic algorithm outputs the maneuver decision values, which serve as the final decision adopted by the blue side.

To ensure rapid convergence of the genetic algorithm, the population size is set to 50(p_1_–p_50_), and each chromosome is encoded as a real number containing three genes corresponding to overload in three directions. The fitness function is defined as the sum of the advantages of one maneuver of the blue side over the seven typical maneuvers of the red side after a single decision step.

When employing genetic algorithms for optimization, a new population is formed through selection, crossover, and mutation operations. Through continuous iteration, this newly generated population evolves towards higher fitness values, with the individual exhibiting the highest fitness ultimately output as the final result of the optimization search.

1. Selection

During the selection process, individuals in the population are first sorted by their fitness from high to low, ranked from 1 to 50. The top 10 individuals (p_1_–p_10_) are retained for the next generation, while individuals numbered 11–40 (p_11_–p_40_) undergo crossover operations to generate 30 new individuals. Additionally, 10 individuals(p_41_–p_50_) are randomly selected for mutation operations.

2. Crossover

During crossover operations, 30 chromosomes are randomly paired, and a gene is randomly selected for position exchange between the paternal and maternal parents in each pair.

3. Mutation

During mutation operations, a gene with a value of *c* is randomly selected, and a mutation is performed within a uniform distribution range of [*c* − 0.5, *c* + 0.5].

The algorithm of opponent UAV decision-making is shown in Algorithm 1.
**Algorithm 1:** Genetic Algorithm Optimizing Matrix Game**Initialization**: Initialize a set containing 7 typical maneuver decisions as {a_1_…a_7_}, an advantage matrix adv_m,n_ filled with zeros.**Matrix Game**:**for** the blue side select *a*_i_ = a_1_…a_7_
**do**: **for** red side select *a*_j_ = a_1_…a_7_ **do**:  Calculate the advantage value *adv*_i,j_ after one decision step  Fill the value into the advantage matrix adv_m,n_ **end for**
**end for**Calculate the row sum and choose the action A* corresponding to the maximum value**Genetic Algorithm:**Build a population of size 50 centered around decision A***for** iteration = 1…20 **do:** Sort individuals in the population based on fitness, numbered p_1_–p_50_ Select p_1_ to p_10_ to directly enter the next generation Select p_11_ to p_40_ for crossover; the offspring enters the next generation Select p_41_ to p_50_ for mutation as the next generation Calculate the fitness of each individual in the next generation population **end for**Select the individual with the highest fitness as the final decision

## 4. Decision-Making Approach Based on the Transformer Network and Deep Reinforcement Learning

**Figure 3:**
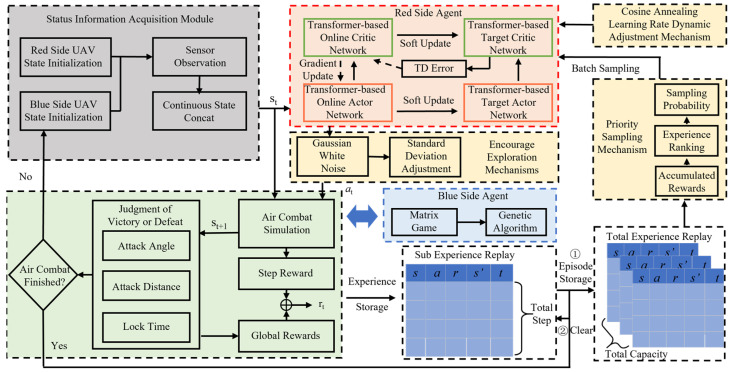
Maneuver decision-making approach based on DRL and the Transformer network.

The structure of the proposed maneuver decision-making approach is shown in Figure 3, which includes the red and blue UAV agents, the air combat environment, and the adopted performance enhancement measures. The red UAV agent is built based on the AC framework by incorporating a Transformer network for both the actor and critic components. The blue UAV agent adopts the approach combining the matrix game and genetic algorithm to make the maneuvering decision. The air combat environment comprises a UAV simulation based on kinematic equations and a victory judgment system. This system provides state and reward feedback to the UAV agents. Also, three measures to improve the performance of the red UAV agent’s decision are given, which are encourage exploration, priority sampling, and learning rate dynamic adjustment.

### 4.1. Reward Function Design

The goal of DRL is to learn a policy, $\pi_{\theta }$, parameterized by *θ*, to maximize the expected cumulative discounted reward defined as follows:

(8) $$\max\;J(\theta )=\max E[\sum_{t=0}^{T}\gamma^{t}r_{t}],\gamma \in (0,1),$$

where *γ* is the discount factor that discounts future rewards to the current time step, and $r_{t}$ represents the reward obtained at time step *t*.

The goal of close-range air combat is to achieve an advantageous attacking position. This occurs when the opponent is within the weapon’s attack angle and the UAV maintains a comparable speed to counter evasive maneuvers. Meanwhile, in order to deal with the poor convergence performance caused by sparse rewards in DRL, a reward function is designed by combining the local and global rewards. The reward function of the UAV agent at time *t* is defined as follows:

(9) $$r_{t}=\alpha r_{l}+(1-\alpha )r_{g},$$

where α is the weight and $r_{l}$ and $r_{g}$ represent the local reward and global reward, respectively. 

The local reward refers to the process of reward to guide UAVs to occupy an advantageous position. The global reward refers to the resulting reward related to the final combat result, and it encourages the UAV agent to win the combat from a global perspective.

The local reward is represented as follows:

(10) $$r_{l}=\alpha_{1}r_{\varphi }+\alpha_{2}r_{v},\;s.t.\;\alpha_{1}+\alpha_{2}=\alpha$$

where $\alpha_{1}$ and $\alpha_{2}$ are the weights for the local angle reward and the local speed reward, respectively. The local angle reward $r_{\varphi }$ and local speed reward $r_{v}$ are designed as follows:

(11) $$r_{\varphi }=\left\{\begin{array}{ll} 20, & 0^{\circ }\leq \varphi^{r}\leq 5^{\circ }\\ 10, & 5^{\circ }<\varphi^{r}\leq 15^{\circ }\\ 5, & 15^{\circ }<\varphi^{r}\leq 30^{\circ }\\ 1, & 30^{\circ }<\varphi^{r}\leq 60^{\circ }\\ -30, & 60^{\circ }<\varphi^{r}\leq 180^{\circ } \end{array}\right.,$$

(12) $$r_{v}=\left\{\begin{array}{ll} 10, & 0\leq |v^{r}|-|v^{b}|\leq 10\\ 8, & 10<|v^{r}|-|v^{b}|\leq 30\\ 5, & |v^{r}|-|v^{b}|>30\\ 3, & -10\leq |v^{r}|-|v^{b}|<0\\ 1, & -30\leq |v^{r}|-|v^{b}|<-10\\ -20, & |v^{r}|-|v^{b}|<-30 \end{array}\right.,$$

Equation (11) defines the angle reward function, which ensures that the blue UAV remains within the red UAV’s attack angle range. According to Figure 2, a smaller velocity leading angle ($\varphi^{r}$) for the red UAV indicates that it is positioned behind the blue UAV, thereby meeting the attack angle condition specified in Equation (2). Equation (12) defines the speed reward function, which aims to make the speed of the red UAV close to the blue UAV. On the one hand, during turning maneuvers in adversarial training, a lower speed is required to achieve a smaller turning radius, resulting in a smaller velocity leading angle. On the other hand, to satisfy the attack distance requirement, a higher speed compared to the blue UAV is encouraged to close the distance.

Also, the global reward is represented as follows:

(13) $$r_{g}=\left\{\begin{array}{ll} 5000, & victory\\ -5000, & defeat\\ 0, & draw \end{array}\right.$$

where the above result is determined by Equation (2).

### 4.2. The Transformer-Based Actor and Critic Networks

A Transformer network is an efficient model for handling sequential data with temporal dependencies, and it is widely applied in fields including machine translation, text summarization, and so on. The core of the Transformer network is the self-attention mechanism, which computes attention weights for different positions in the input sequence. The key component of self-attention is scaled dot-product attention, and it is described as follows:

(14) $$Attention(Q,K,V)=softmax(\frac{Q\cdot K^{T}}{\sqrt{d_{k}}}+M)\cdot V,$$

where *Q*, *K,* and *V* represent the query matrix, key matrix, and value matrix, respectively. $d_{k}$ denotes the dimensions of both the query matrix and the key matrix, and *M* is a lower triangular matrix used for computing masked self-attention. Especially, the query (*Q*), key (*K*), and value (*V*) matrices are linear transformations derived from the input sequence. Assuming the input sequence is X, the matrices are computed as $Q=W_{Q}*X$, $K=W_{K}*X$, $V=W_{V}*X$, where $W_{Q},\;W_{K}$, and $W_{V}$ are learnable weight matrices.

The multi-head attention mechanism consists of multiple sets of dot-product self-attention. Each self-attention mechanism enables the model to focus on a specific feature. Meanwhile, multi-head attention allows interaction between multiple heads to enhance its representational capacity. By concatenating matrices with different attention focuses, it obtains the features of different inputs. The multi-head attention is described as follows:

(15) $$\begin{array}{l} head_{i}=Attention(Q_{i},K_{i},V_{i})\\ MultiHead(Q,K,V)=concat(head_{1},\ldots ,head_{i})\cdot W^{0} \end{array}$$

where $W^{0}$ is a matrix of linear transformation coefficients.

**Figure 4:**
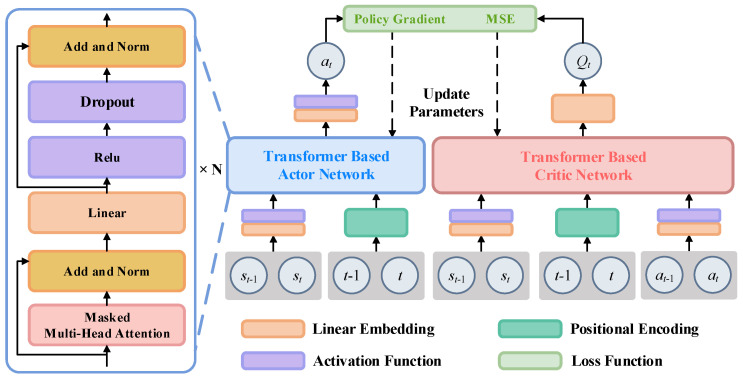
The structure of actor and critic networks is based on the GPT model.

The structure of actor and critic networks based on the Transformer-based GPT model is shown in Figure 4, and both the networks consist of multi-head attention, residual connection, layer normalization, feedforward neural network, and other structures. In the actor network, a linear layer and position encoding layer are used to embed the states and to encode time instants, respectively, and then through the layers including dropout, masked multi-head attention, and other layers, the action output is obtained. Similar to the actor network, the inputs of the critic network are states, actions, and instants, and after passing through the linear layer, encoding layer, masked multi-head attention, and other layers, the state–action value *Q*(*t*) is obtained.

In the structure of actor and critic networks based on the GPT model, positional encoding plays a crucial role in addressing the position order problem in sequence data. Unlike traditional recurrent neural networks (RNNs), which inherently handle the sequential order of data, the Transformer architecture is entirely based on the self-attention mechanism, which is position-agnostic. Therefore, positional information needs to be explicitly injected into the model.

Positional encoding is typically achieved by adding a sequence of vectors—one for each position in the input sequence—to the input state embeddings. This allows the model to distinguish between states at different steps. Here, a commonly used method involving generating positional encodings using sine and cosine functions is adopted. Specifically, each position’s encoding is calculated as follows:

(16) $$\begin{array}{l} PE(pos,2i)=sin(\frac{\mathrm{pos}}{10000^{2i/d}})\\ PE(pos,2i+1)=\cos(\frac{\mathrm{pos}}{10000^{2i/d}}) \end{array}$$

where pos is the position of the time step, *i* is the index of the vector, and d is the dimension of the encoding vector. 

### 4.3. Priority Sampling Mechanism Based on Episode Experience Replay

In the Transformer network, the continuous time series data of the state–action pair are used to train the network. However, in the traditional DRL, the experience replay stores one time instant of state–action data as an experience, which cannot be used to train the Transformer network. Furthermore, even if several experience samples are drawn, it is hard to determine the time order of these samples and meanwhile guarantee these experience samples have continuity in time and space dimensions, leading to incorrect training of the Transformer network. Thus, it is necessary to store the state–action time series as an experience sample in the experience pool, which is convenient for experience sampling to train the Transformer-based actor and critic network. Also, the random sampling approach in experience replay cannot ensure that the high-quality experience samples are efficiently used and learned, which has an influence on the convergence speed and quality of the DRL algorithm. This problem will worsen in the Transformer-based actor and critic network. Therefore, it is necessary to design an effective sampling approach in experience replay to improve the learning efficiency of the proposed DRL method.

To deal with the above problems, an episode-based replay memory is presented, and based on it a priority sampling approach is proposed. The episode-based replay memory collects an episode of state–action transitions as a single experience, which is stored in the sub-memory. When an episode of air combat is finished, the episode experience in the sub-memory is put in the replay memory and then the sub-memory is clear. Also, in the priority sampling approach, each episode-based experience is arranged in order based on cumulative reward, and the sampling probability is proportional to the sort priority of experiences. This approach guarantees that the good quality experience sample can be drawn in a larger probability, improving the convergence speed and quality of the proposed DRL algorithm. 

**Figure 5:**
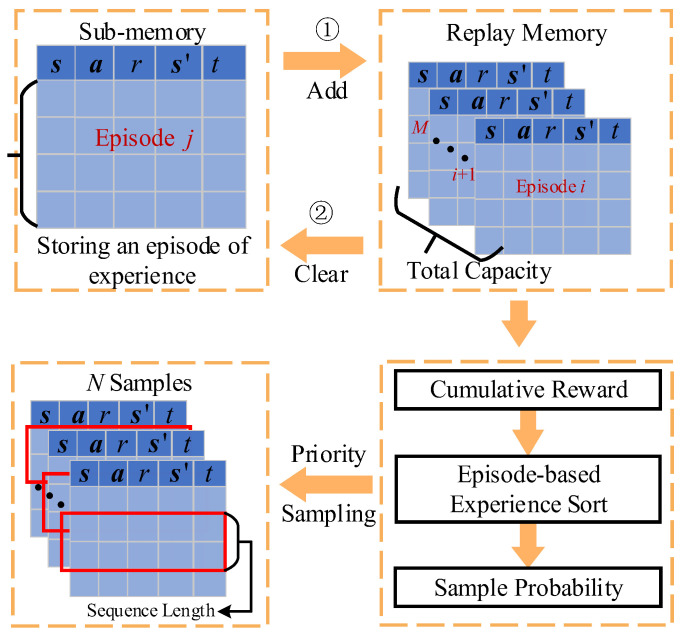
Priority sampling approach based on episode-based experience replay.

Figure 5 illustrates the episode-based replay and priority sampling mechanism. Firstly, the sub-experience memory with the capacity defined as *B*_1_ is designed, which is used to store the experiences generated in an episode. If the end time of an episode is less than the maximum simulation time, *t*_max_, then the remaining time steps are padded with zeros. When an episode is finished, an episode of experiences is added to the total replay memory. Meanwhile, the sub-memory is cleared and prepared to store the next episode of experiences. The capacity of total replay memory is defined as *B*. When the total replay memory is full, the first-in-first-out rule is used to update the replay memory. The introduction of episode-based replay memory can improve the sampling efficiency since the continuous time samples are required to train the Transformer-based network.

When the replay memory is full, the priority sampling approach is used to obtain the sample from the replay memory to train the actor and critic network. The cumulative reward of each episode-based experience in the replay memory is first calculated, and then all experiences are sorted in ascending order by cumulative reward. As a consequence, the experience with the largest cumulative reward has the maximum sort number. The sampling probability of each experience is computed as follows:

(17) $$P_{j}=j/\sum_{j=1}^{M}j,$$

where *j* represents the sort number, *M* is the number of experiences in the reply memory, and *Pj* is the sampling probability. *N* samples are drawn from the replay memory based on the sampling probability. For each sample, continuous time series with sequence lengths of *C* (i.e., $t_{i},t_{i+1},\cdots ,\;t_{i+c-1}$) are collected in random sampling to constitute a batch of samples. This priority sampling approach can collect higher reward experience samples for training the actor and critic networks, which can improve the learning efficiency and accelerate the algorithm’s convergence.

### 4.4. Dynamic Learning Rate Adjustment for Stable Training

For the Transformer-based network, it is necessary to adjust the learning rate as the training process goes on. At the initial training stage, a relatively large learning rate is usually used in order to adjust network weights quickly by adopting gradient descent optimization. As the training process goes on, the network has a better nonlinear mapping capability, and then it is more important to focus on the precision of the network output. This means that at the late stage, the network should use a relatively small learning rate to guarantee the network output converges to the optimal solution. Therefore, the dynamic learning rate adjustment by using a cosine annealing schedule is designed as follows:

(18) $$lr_{cur}=lr_{min}+\frac{1}{2}(lr_{max}-lr_{min})\cdot (1+\cos(\frac{T_{cur}}{T_{max}}\pi )),$$

where *lr_cur_* is the current learning rate, *lr_max_* and *lr_min_* are the set maximum and minimum learning rates, *T_cur_* represents the current number of training episodes, and *T_max_* is the total number of training episodes. By analyzing Equation (18), it is seen that the learning rate decreases as the training process continues, and the descent speed of the learning rate is small at the initial and late training stages but large at the middle training stage. In other words, the network gets close to the optimal solution quickly with a large learning rate at the initial stage, and after that, the learning rate decreases quickly at the middle state and then uses a small learning rate to search for the optimal solutions at the late stage. This is beneficial for stabilizing the reinforcement learning training process and obtaining a good decision result.

### 4.5. Trade-Off Between Exploration and Exploitation

A common challenge in deep reinforcement learning (DRL) is balancing the trade-off between exploration and exploitation. Exploration means trying actions that improve the model, whereas exploitation means behaving in an optimal way given the current model. In this paper, the Deep Deterministic Policy Gradient algorithm is adopted, and the actor network outputs deterministic actions. The actor network parameters are initialized randomly, but the actor outputs make little difference to various inputs at the beginning of training. Therefore, the actor outputs are not beneficial for exploring the potential good maneuver strategy. To encourage exploration, Gaussian white noise is added to the actor output in order to encourage exploration, which is expressed as follows:

(19) $$\left\{\begin{array}{c} \begin{array}{l} a_{t}=\mu \left(s_{t};\theta \right)+\eta \\ \eta \sim N(0,\sigma^{2}) \end{array}\\ a_{t}\sim clip\left(N_{min},N_{max}\right) \end{array}\right.,$$

where $a_{t}$ represents the executed action; $\mu \left(s_{t};\theta \right)$ denotes the action output by the actor network; η is the Gaussian white noise and its mean and standard deviation are 0 and σ; and *clip*() represents the truncation function that determines the action values in the range of $\left(N_{min},N_{max}\right)$.

Based on Equation (19), it is seen that the noise has an influence on the degree of exploration. At the initial stage of training, the noise standard deviation is set relatively high to encourage efficient exploration of the action space. As the training goes on, the standard deviation of noise decreases gradually in order for the agent to achieve a balance between exploration and exploitation. At the end stage, the standard deviation is maintained at a specified small value in order to make the agent exploit the optimal maneuver strategy given the memory. Thus, inspired by the epsilon-greedy policy, the noise standard deviation is controlled as follows:

(20) $$\sigma =\left\{\begin{array}{cc} \sigma_{init}+\frac{\left(\sigma_{init}-\sigma_{end}\right)}{T_{end}}T & 0\leq T\leq T_{end}\\ \sigma_{end} & T_{end}<T\leq T_{max} \end{array}\right.,$$

where $\sigma_{init}$ and $\sigma_{end}$ are the initial and end values of the noise standard deviation; T and $T_{max}$ are the current and maximum number of episodes; $T_{end}$ represents the episode number; and the noise standard deviation is equal to the end value. In the last training stage (i.e., $T_{end}<T\leq T_{max}$), the noise standard deviation is set to $\sigma_{end}$, which is not equal to zero. This prevents the experience samples in the replay memory from becoming overly homogeneous and subsequently protects the critic network from overfitting. Therefore, by adding Gaussian random noise to the actor output and controlling its standard deviation, the balance between exploration and exploitation is achieved.

### 4.6. Network Training and Policy Update

Once enough experience samples are stored in the replay memory, the priority sampling mechanism is used to collect a batch of samples. These samples are used for training both the actor and critic networks, and the network parameters are updated through gradient descent and error backpropagation. The proposed maneuver decision-making approach introduces the Transformer network in the DDPG algorithm, which contains four Transformer-based networks: the online and target actor networks with the parameters *θ* and *θ*′, and the online and target critic networks with the parameters ω and $\omega^{\prime }$. The online actor network takes the state $s_{t}$ as input and outputs the maneuvering action $a_{t}$. The online critic network takes the joint state–action $\left(s_{t},a_{t}\right)$ as input and outputs the evaluation value *Q* for the joint state–action pair.

A continuous time experience sample with sequence length *l* is defined as follows:

(21) $$\begin{array}{ll} Sampl_{i} & =\left[S_{t_{i}-l+1:t_{i}}^{i},\;A_{t_{i}-l+1:t_{i}}^{i},\;R_{t_{i}-l+1:t_{i}}^{i},\;S_{t_{i}-l:t_{i}+1}^{i}\right]\\ & =\left[\begin{array}{cccc} s_{t_{i}-l+1} & a_{t_{i}-l+1} & r_{t_{i}-l+1} & s_{t_{i}-l+2}\\ s_{t_{i}-l+2} & a_{t_{i}-l+2} & r_{t_{i}-l+2} & s_{t_{i}-l+3}\\ \vdots & \vdots & \vdots & \vdots \\ s_{t_{i}} & a_{t_{i}} & r_{t_{i}} & s_{t_{i}+1} \end{array}\right], \end{array}$$

where $S_{t_{i}-l+1:t_{i}}^{i}$, $A_{t_{i}-l+1:t_{i}}^{i}$, and $R_{t_{i}-l+1:t_{i}}^{i}$ are the continuous time sequence of state, action, and reward, and $t_{i}$ is a randomly selected time instant in an episode satisfying $l\leq t_{i}\leq T_{max}$. The *N* samples are defined as a batch, which is given as follows:

(22) $$Batch=\left\{Sampl_{1},\;Sampl_{2},\;\cdots ,\;Sampl_{N}\right\},$$

For each sample, the target *Q* value for a time $t_{i}$ is calculated as follows:

(23) $$Q_{t_{i}}^{target}=r_{t_{i}}+\gamma \cdot Q^{\prime }(S_{t_{i}-l:t_{i}+1}^{i},\mu^{\prime }(S_{t_{i}-l:t_{i}+1}^{i};\theta^{\prime });\omega^{\prime })$$

where $Q^{\prime }$ represents the *Q* value generated by the target critic network, and $\mu^{\prime }$ represents the use of the target actor network. By minimizing the mean-square-error loss function defined in Equation (24), the online critic network parameters are updated.

(24) $$Loss_{C}=\frac{1}{N}{\sum_{i}\left(Q_{i}^{target}-Q(S_{t_{i}-l+1:t_{i}}^{i},A_{t_{i}-l+1:t_{i}}^{i};\omega )\right)}^{2}$$

The online actor network is used to produce the maneuvering strategy for maximizing the expected cumulative discounted reward. The policy gradient of the actor network is computed as follows:

(25) $$\begin{array}{ll} \nabla_{\theta }J(\theta ) & =\nabla_{a}Q(s,a)\cdot \nabla_{\theta }\mu (s)\\ & \approx \frac{1}{N}\sum_{i=1}^{N}\nabla_{a}Q(S_{t_{i}-l+1:t_{i}}^{i},A_{t_{i}-l+1:t_{i}}^{i};\omega )\nabla_{\theta }\mu (S_{t_{i}-l+1:t_{i}}^{i};\theta ), \end{array}$$

Based on Equations (24) and (25), the parameters of the online critic and actor networks are updated as follows:

(26) $$\begin{array}{c} \theta \leftarrow \theta +lr_{cur}\cdot \nabla_{\theta }J(\theta )\\ \omega \leftarrow \omega -lr_{cur}\cdot \nabla_{\omega }Loss_{C} \end{array},$$

where τ is the soft update parameter to control the update degree.

The algorithm of the proposed approach is shown in Algorithm 2.
**Algorithm 2:** Decision-making approach based on the Transformer network and deep reinforcement learning.**Initialization**: Initialize a sub-memory ***R***_1_ with capacity B_1_ and episode-based replay memory ***R*** with capacity B; online actor and critic networks based on the Transformer network with weights θ, ω; target actor and target critic networks with weights $\theta^{\prime }=\theta ,\;\omega =\omega^{\prime }$; and a random process N with a mean of 0 and an initial standard deviation of $\sigma_{init}$
**for** episode = 1,2,…$T_{max}$ **do**: Initialize continuous time sequence of state $s_{1}$ in air combat **for** t = 1,2,…, $t_{max}$ **do**:  Select action $a_{t}=\mu (s_{t};\theta )+N$ according to the online actor network   Execute action $a_{t}$, get a reward $r_{t}$ and a new state $s_{t+1}$
  Store a transition with [$s_{t},a_{t},r_{t},s_{t+1}$] in sub-memory ***R***_1_  If episode-based replay memory ***R*** is full:   Sample a minibatch of N from ***R*** with priority sampling weight *P_j_*   Compute $Q_{t_{i}}^{target}=r_{t_{i}}+\gamma \cdot Q^{\prime }(S_{t_{i}-l:t_{i}+1}^{i},\mu^{\prime }(S_{t_{i}-l:t_{i}+1}^{i};\theta^{\prime });\omega^{\prime })$
   Update the online critic network by minimizing the loss:$Loss_{C}=\frac{1}{N}{\sum_{i}\left(Q_{i}^{target}-Q(S_{t_{i}-l+1:t_{i}}^{i},A_{t_{i}-l+1:t_{i}}^{i};\omega )\right)}^{2}$   Update the online actor network by using the policy gradient:$\nabla_{\theta }J(\theta )=\frac{1}{N}\sum_{i=1}^{N}\nabla_{a}Q(S_{t_{i}-l+1:t_{i}}^{i},A_{t_{i}-l+1:t_{i}}^{i};\omega )\nabla_{\theta }\mu (S_{t_{i}-l+1:t_{i}}^{i};\theta )$   Update the target actor and target critic networks:$\begin{array}{l} \theta^{\prime }\leftarrow \tau \cdot \theta +(1-\tau )\cdot \theta^{\prime }\\ \omega^{\prime }\leftarrow \tau \cdot \omega +(1-\tau )\cdot \omega^{\prime } \end{array}$  If air combat is finished:   break **end for**
 Put the sub-memory to episode-based replay memory and clear the sub-memory Decay the standard deviation of a random process and the learning rate of networks**end for**

## 5. Simulation Experiment

To verify the feasibility and stability of the algorithm, simulation experiments were conducted to perform one-on-one close-range air combat maneuver decision-making. These experiments enabled the agent to seize advantageous attack positions during air combat and achieve the desired combat objectives. In order to determine whether a priority sampling mechanism is used, whether a dynamic learning rate adjustment is used, and the size of the set sequence length, and to verify the feasibility of the algorithm, the situation of the opposing sides is transparent during the process; that is, the opposing sides can obtain information from each other. In addition, to verify the superiority of using the Transformer network architecture, a comparison was made between the Transformer network used in this paper and the deep reinforcement learning method using a fully connected network. Corresponding experimental scenarios were set up in the case of losing opponent information with a 10% probability.

### 5.1. Simulation Experiment Parameters

The simulation parameters for a one-to-one close-range air combat environment are shown in Table 1.

All the simulation experiments in this paper were conducted on a PC configured with Intel i7-11700, 32 GB of memory, and running on the Win10 operating system. The experiments were compiled and developed using Python 3.8 and PyCharm 2021.3.

The network parameters used in this paper and the necessary parameter configurations in deep reinforcement learning are shown in Table 2 and Table 3.

### 5.2. Performance Analysis of the Improvement Mechanisms

**Figure 6:**
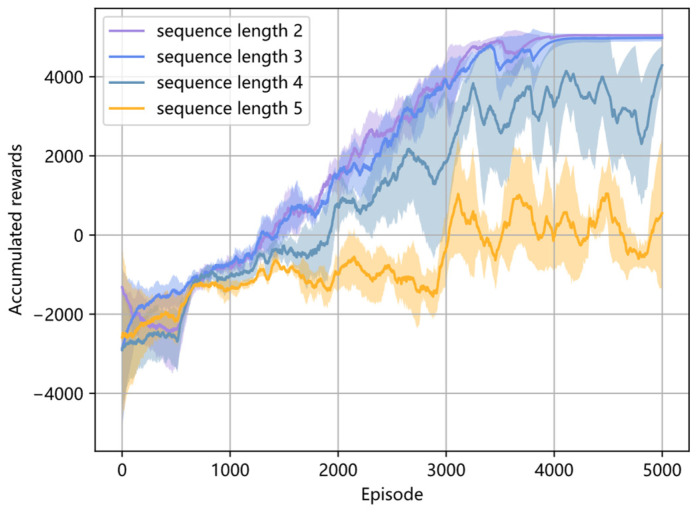
Accumulated reward curves with different sequence lengths.

Firstly, the influence of different sample sequence lengths on the performance of maneuver decision-making is analyzed. The sample sequence lengths *l* are set as 2, 3, 4, and 5, respectively, and the accumulated reward curves of different sequence lengths are shown in Figure 6. It illustrates that the model is trained well with a large accumulated reward when the sequence length is set as 2 and 3. Moreover, as the sequence length increases, the Transformer network faces greater difficulty in capturing spatial–temporal relationships, leading to unstable training results, as shown in Figure 6. Since air combat is a Markov decision process, when the current information is lost, the previous one or two time instant information is the most reliable information used to estimate the current information. Thus, the result of selecting a sequence length of 2 and 3 is reasonable.

Furthermore, Table 4 gives the winning rate with different sequence lengths in the last 2000 training episodes. It shows that the highest average winning rate with the smallest standard deviation is obtained when the chosen sequence length is 2, and a sequence length of 3 comes second. The average winning rate decreases rapidly when the sequence length is larger than 3. Therefore, the sequence length is set as 2 in the following simulation.

**Figure 7:**
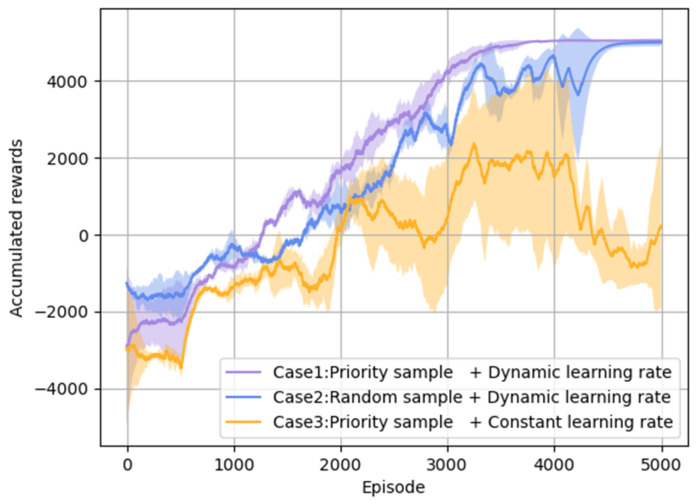
Cumulative reward curves for DRL model training in three cases.

Then, the performance influence of introducing the improvement mechanisms is analyzed in Figure 7. Three cases are compared as follows: Case 1, priority sampling +dynamic learning rate adjustment; Case 2, random sampling +dynamic learning rate adjustment; and Case 3, priority sampling +constant learning rate (*lr* = 0.0001). The episode cumulative reward curves in the above three cases are present in Figure 6, which are obtained by several experiments with different random seeds. By comparing the results of Case 1 and Case 2, it is seen that the training process converges faster when using priority sampling. This result indicates that the proposed priority sampling method can improve the DRL model training convergence. By comparing the reward curves of Case 2 and Case 3, it is seen that the model is convergent to the stable status with a large stable cumulative reward, which is rapidly acquired by using the dynamic learning rate. On the contrary, by using the constant learning rate, the model training is unstable and the agent performs poorly in combat since the large learning rate fails to converge to a stable optimal or sub-optimal maneuvering strategy at the last training stage.

The average winning rate and its standard deviation in the last 2000 training episodes via multiple experiments are given in Table 5. It shows that the agent has the highest average winning rate with the smallest standard deviation by using priority sampling and dynamic learning rate adjustment. Thus, it also proves that the DRL model training can be more effective and stable by using the improvement mechanisms. 

### 5.3. Performance Comparison of Different DRL Approaches

Here, we compare the maneuver decision-making performance of the proposed method, the LSTM network, and the traditional DDPG and PPO methods.

**Figure 8:**
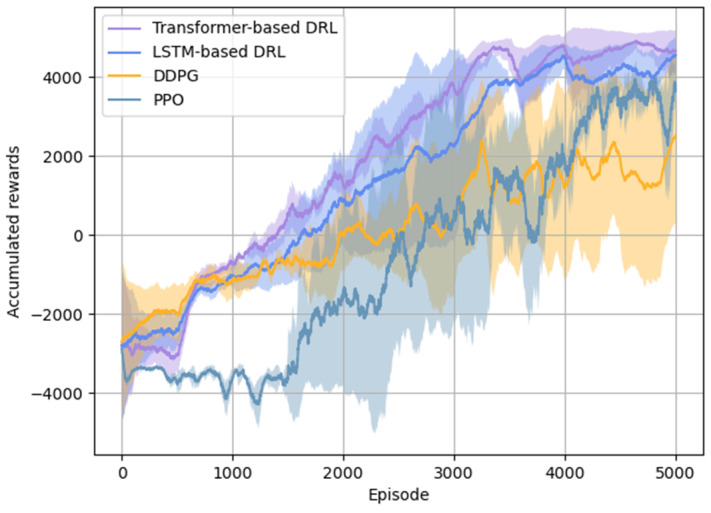
Comparison of accumulated rewards between the Transformer and LSTM networks and the DDPG and PPO methods.

Figure 8 compares the DDPG method based on the Transformer network and the LSTM network (both with a sequence length of 2) with the DDPG and PPO methods using fully connected neural networks (with a sequence length of 1). The curves in the figure show that, even in the presence of information loss, the Transformer network, designed for processing time series data, outperforms the LSTM network during training. In contrast, the fully connected network, which only uses the current state as input, fails to make high-quality decisions when information is lost, resulting in non-convergence of the training outcomes for both the DDPG and PPO methods.

Table 6 compares the winning rates from the last 2000 training process using the three methods mentioned above under conditions of information loss. The data indicate that the Transformer network effectively captures temporal dependencies, achieving higher winning rates with smaller standard deviations, resulting in a more stable training process. The LSTM network performs slightly worse than the Transformer network but still outperforms the fully connected network. In contrast, the fully connected network exhibits a lower average winning rate, larger standard deviations, and unstable training, leading to a lack of convergence.

Table 7 compares the time consumption for single-step decisions using the Transformer network, the fully connected neural network, the LSTM network, and matrix game methods, as previously described in this article. The matrix game method requires the longest time, making it difficult to meet the real-time demands of air combat. The Transformer network takes 0.78 ms longer per single step than the fully connected network but achieves a winning rate exceeding 44.1% in scenarios involving opponent information loss. Due to the sequential input processing and the complex gating mechanisms, the LSTM network exhibits significantly higher time costs per step compared to the parallelized Transformer network. This demonstrates that, while satisfying the real-time requirements of air combat, the Transformer network effectively enhances the winning rate in situations with opponent information loss, validating the superiority of the proposed method.

### 5.4. Adversarial Training

**Figure 9:**
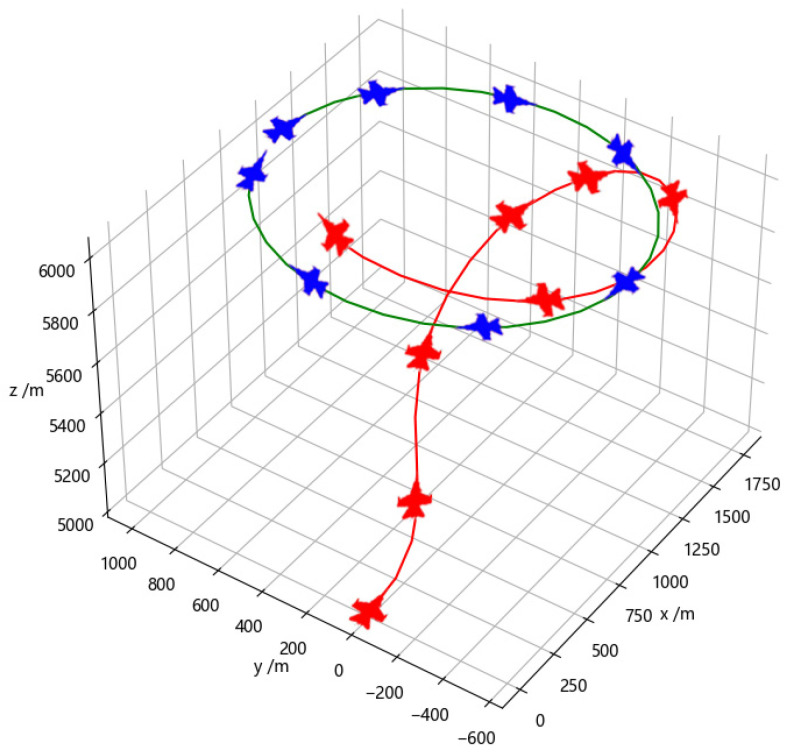
Adversarial training with the Transformer network.

Figure 9 depicts the confrontation between agents trained using the Transformer network in a specific episode. In the early stage of the episode, the red agent chooses to decelerate and climb to the left to gain an angular advantage, while the blue one continues turning right to achieve an attacking angle advantage. The red agent anticipates the continued right turn of the blue opponent by analyzing its previous right-turn trajectory. Therefore, the red agent takes an early right turn and climb action to gain angular and distance advantages. In the middle stage, with both sides at similar altitudes, the red agent adopts a defensive stance against the blue agent and attempts to escape the tail chase. The blue agent continues turning right to evade while the red agent chooses to keep decelerating to achieve a smaller turning radius and quickly gain an angular advantage. However, at this point, the blue agent’s higher speed causes the distance between them to increase rapidly, and the red agent loses the attack lock. In the end, the red agent obtains a significant angular advantage and enters a pursuit position against the blue agent. It chooses to maintain acceleration, continuously reducing the distance between them. Eventually, meeting the attack conditions, the red agent successfully locks and attacks the blue agent, resulting in a victory for the red side in this episode.

**Figure 10:**
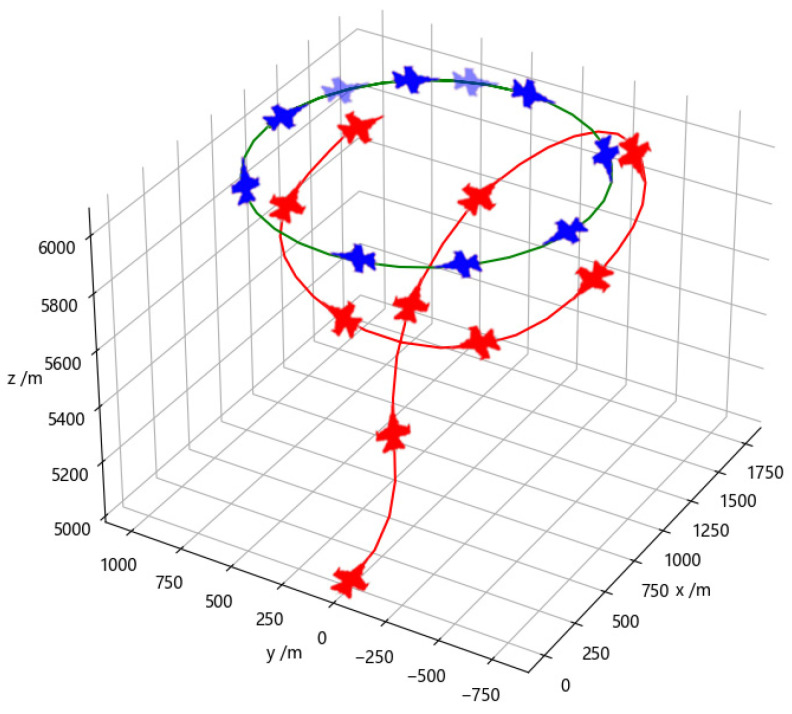
Adversarial training with the fully connected network.

Figure 10 illustrates the adversarial process of a specific episode during training using a fully connected network. In contrast to training with the Transformer network, the red agent still chooses to accelerate in the middle stage of the episode, despite the loss of information about the blue opponent. This prevents the red agent from engaging in a one-circle confrontation with a smaller turning radius, resulting in missing the attack opportunity. In the late stage, by adjusting its own maneuver decisions during the one-circle engagement, the red agent eventually establishes a tail chase against the blue agent. After meeting the attack conditions, the episode is determined as a victory for the red side. This demonstrates a difference in decision-making between the Transformer network and the fully connected network training, with the latter adapting its maneuver decisions during the engagement to overcome the challenges posed by the loss of opponent information.

**Figure 11:**
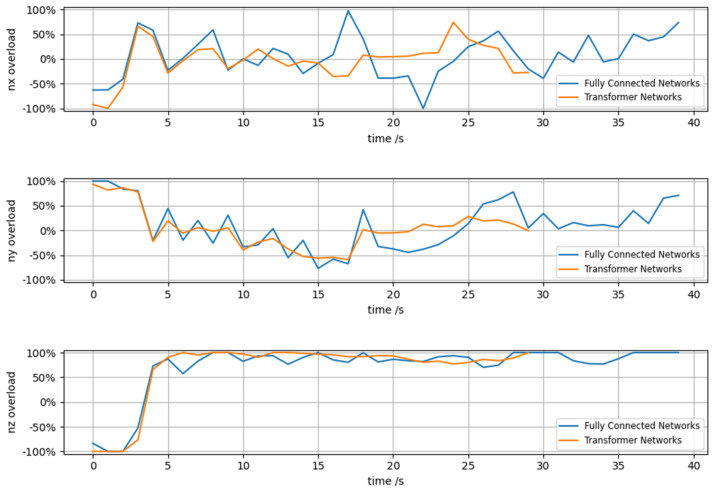
Comparison of overloading in all directions of the red side.

Figure 11 shows the comparison of the three-direction overload changes of the red agent during adversarial training using the Transformer and fully connected networks in Figure 9 and Figure 10. It can be clearly seen from the three-direction overload in the figure that at the 29th second, the agent using the Transformer network completed the air combat ahead of schedule, which is faster than using a fully connected network to complete the strike. The overload changes of the agent using the Transformer network are relatively smooth, with fewer abrupt mutations. In contrast, the agent using the fully connected network exhibits larger overload variations and tends to output edge-controlling decision instructions.

Table 8 compares the energy consumption for each overload of agents using the Transformer network and the fully connected network under the control instructions shown in Figure 11. Energy is defined as the area enclosed by the overload and the time axis in all directions. This table demonstrates that UAVs utilizing Transformer networks can defeat opponents with less energy and in a shorter time, leading to an advantage in air combat. This further confirms the superiority of the method proposed in information loss environments.

## 6. Conclusions

This paper addresses the issue of UAVs facing difficulties in obtaining opponent information, which affects the quality of autonomous decision-making and the win rate of air combat. By introducing Transformer networks into deep reinforcement learning algorithms, we aim to capture the dependency relationships between sequence information and maintain high-quality decision-making capabilities, even in the absence of information. In order to address the issues of poor training stability, insufficient training samples, and low sampling efficiency caused by the introduction of a Transformer network, dynamic adjustment of the learning rate, the encouragement of exploration mechanism, episode experience replay, and the priority sampling mechanism were used, respectively, effectively improving training stability and efficiency. A large number of simulation results show that the proposed method exhibits higher decision quality and higher win rate compared to traditional fully connected networks, further verifying the superiority of the proposed method.

## Figures and Tables

**Table 1:** Close-range air combat simulation parameters.

Name	Symbol	Value
Simulation step/s	*t_s_*	0.1
Decision step/s	*t_p_*	1
Simulation time/s	*t_max_*	60
Simulated minimum speed/(m·s^−1^)	*V_min_*	100
Simulated maximum speed/(m·s^−1^)	*V_max_*	300

**Table 2:** Actor and critic network parameters.

Parameter	Value	Parameter	Value
State vector dimension	9	Linear connection layer dimension	128
Action vector dimension	3	Actor output activation function	Tanh
Linear embedding layer dimension	64	Critic output activation function	-
Dropout layer inactivation rate	0.1	Maximum learning rate lrmax	0.001
Multiple attention heads	16	Minimum learning rate lrmin	0.00001
*Q K V* matrix dimension	64	Optimizer	Adam

**Table 3:** DRL model training parameters.

Parameter	Value
Discount factor γ	0.9
Total replay memory capacity B (episode)	512
Sub-replay memory capacity B_1_ (piece)	60
Number of batch sampling *N* (piece)	256
Soft update parameter τ	0.05

**Table 4:** Comparison of winning rates for different sequence lengths.

Sequence Length	Average Winning Rate	Standard Deviation of Winning Rate
2	98.0%	0.040
3	96.1%	0.068
4	76.1%	0.156
5	27.1%	0.158

**Table 5:** Winning rate for three cases.

Case	Average Winning Rate	Standard Deviation of Winning Rate
Case 1: priority sampling + dynamic learning rate	98.6%	0.026
Case 2: random sampling + dynamic learning rate	89.5%	0.148
Case 3: priority sampling + constant learning rate	35.7%	0.242

**Table 6:** Comparison of winning rates for three deep reinforcement learning methods.

Method	Average Winning Rate	Standard Deviation of Winning Rate
Transformer	92.3%	0.068
LSTM	83.8%	0.113
DDPG	48.2%	0.135
PPO	86.1%	0.224

**Table 7:** Comparison of time consumption for single-step decision-making.

Method	Single-Step Decision Duration (ms)	Time Consumption Percentage (%)
Transformer	0.95	4.929
DDPG/PPO	0.17	0.911
LSTM	2.38	12.48
Genetic Algorithm Optimizing Matrix Game	19.07	100

**Table 8:** Comparison of different methods for controlling energy.

Method	Overload Value and Area Under the Time Axis		
	$n_{x}$	$n_{y}$	$n_{z}$
Transformer	23.2970	36.5637	79.2495
Fully connected network	39.2644	56.7764	102.6854

## Data Availability

Data are contained within the article.
